# Priapism Episode in an Adolescent with Autism Spectrum Disorder Under Antipsychotic Treatment: A Case Report and Literature Review

**DOI:** 10.1192/j.eurpsy.2026.10548

**Published:** 2026-07-31

**Authors:** A. Vazquez Vazquez, G. Villarreal Orellano, E. Morales Vega

**Affiliations:** 1 Unidad de Salud Mental Infanto Juvenil, Hospital Universitario Nuestra Señora de Valme, Sevilla; 2 Hospital Universitario Punta de Europa, Algeciras; 3 Hospital Universitario Jerez de la Frontera, Jerez de la Frontera, Spain

## Abstract

**Introduction:**

Priapism is a rare but potentially serious urological emergency, defined as a prolonged and painful erection unrelated to sexual stimulation. Although uncommon in children and adolescents, it can cause significant morbidity if not promptly recognized and treated. Among pharmacological causes, antipsychotics are relevant triggers, mainly due to alpha-1 adrenergic receptor blockade. In youth with Autism Spectrum Disorder (ASD), atypical antipsychotics such as risperidone, aripiprazole, and olanzapine are frequently prescribed for irritability and behavioral symptoms. Despite widespread use, reports of priapism in this population are scarce, limiting risk estimates and clear management guidance.

**Objectives:**

To present a case of antipsychotic-induced priapism in an adolescent with ASD and review available evidence on risk factors, management, and clinical implications.

**Methods:**

Clinical information was obtained from medical records and caregiver interviews. A narrative literature review was conducted in biomedical databases using terms related to priapism, antipsychotics, children, adolescents and ASD.

**Results:**

A 16-year-old male with ASD, on long-term risperidone (4.25 mg/day), was prescribed olanzapine (2.5mg/day) for insomnia. Four days after daily use of olanzapine, he presented to the emergency department with priapism requiring surgical intervention. Both risperidone and olanzapine were discontinued, and aripiprazole plus benzodiazepines were initiated, leading to favorable clinical outcome without recurrence. Caregivers were counseled on warning signs and the need for urgent medical evaluation.

The literature indicates that priapism risk is linked to alpha-1 affinity: risperidone, quetiapine, ziprasidone, and chlorpromazine are considered higher-risk, while aripiprazole and olanzapine have lower but not negligible risk. Cases with risperidone have been reported even after long-term exposure or in combination with other psychotropics. Aripiprazole, due to its low alpha-1 affinity, is often viewed as a safer alternative. Reports emphasize that priapism can occur without immediate temporal association to treatment initiation, highlighting the need for continuous vigilance. Clinical monitoring should be proactive in ASD, given communication barriers that may delay detection. Professional guidelines stress education of caregivers and urgent referral to urology when symptoms arise.

**Conclusions:**

Antipsychotic-induced priapism in adolescents with ASD is rare but clinically significant. The presented case illustrates the importance of early recognition, prompt discontinuation of the offending agent, and transition to lower-risk alternatives such as aripiprazole. Continuous monitoring, caregiver education, and collaboration between psychiatry, pediatrics, and urology are essential.

**Disclosure of Interest:**

None Declared

